# Selective nitrate removal from aqueous solutions by a hydrotalcite-like absorbent FeMgMn-LDH

**DOI:** 10.1038/s41598-020-72845-3

**Published:** 2020-09-30

**Authors:** Hongguang Zhou, Youlin Tan, Wei Gao, Yue Zhang, Yanmei Yang

**Affiliations:** 1 National Engineering Research Center for Inland Waterway Regulation, Chongqing Jiaotong University, Chongqing, 400074 China; 2 North China Municipal Engineering Design and Research Institute Co. Ltd., Tianjin, 300074 China

**Keywords:** Pollution remediation, Two-dimensional materials

## Abstract

FeMgMn-LDH, a type of potential environmental remediation material, has been synthesized via a co-precipitation method, and its adsorption characteristics for nitrate were investigated in this study. It’s shown that the prepared FeMgMn-LDH is a promising adsorbent for anions removal, which has high buffer capacity (final pH remained between 9 and 10) and high reversibility, and can remove nitrate ions selectively though an anion-sieve effect. The maximum amount of nitrate adsorption is 10.56 N-mg g^−1^ at 25 ℃. The removal rate of nitrate ions can reach 86.26% with the adsorbent dose of 5 g/L in a real water. The competition order of coexisting anions on nitrate adsorption by FeMgMn-LDH is CO_3_^2−^ > PO_4_^3−^ > SO_4_^2−^. The negative values of ΔG^0^ (from − 27.796 to − 26.426 kJ mol^−1^) and ΔH^0^ (− 6.678 kJ mol^−1^) indicate that the nitrate adsorption process on the FeMgMn-LDH is spontaneous and exothermic. The main adsorption mechanisms of nitrate removal from aqueous solutions by FeMgMn-LDH are electrostatic attraction and ion exchange.

## Introduction

Excess nitrate in aquatic environment have become a serious human health risk. High nitrate concentration in drinking water may lead to methemoglobinemia^1^ or blue baby syndrome^2,3^, which could induce the gastric and intestinal cancer^4,5^. The sources of nitrate mainly come from point and nonpoint sources, including urban and agricultural runoff^6^, animal excrement, leakage of septic tank system, refuse landfill leachate^3^ and over-fertilization^7,8^. Because of the low adsorption affinity to soil and high water solubility, nitrate is considered as the most extensive groundwater fomite in the world, which poses a serious threat to the safety of drinking water. Facing the potential health risks, the content of nitrate in drinking water is strictly regulated by the countries all over the world^2^. The highest level of NO_3_-N set by the US Environmental Protection Agency (EPA) in drinking water is 10 mg/L, and that set by China is 20 mg/L. Additionally, NO_3_-N is the main inducement of water eutrophication^9,10^. Therefore, it is urgent need to develop effective methods and materials to remove excess nitrate from water. Reclamation of nitrate ions from water is one of the most serious challenges all over the world.

Nowadays, different methods have been used for nitrate removal, such as adsorption^10,11^, electro-osmosis^12^, catalyzed reduction^13^, membrane filter technology^14^ reverse osmosis^15^ and freezing-melting process^16^. Among these, adsorption has been recognized as one of the most attractive methods for the removal of nitrate from aqueous solutions due to its convenience, easy handling, low budget and simple design. Various adsorbents have been investigated, such as activated carbon^17^, metal oxide hydroxides, activated alumina, modified zeolite, layered double hydroxides^18,19^, slag, fly ash, red mud, crop residues, peat, kaolinite and so on. These kinds of materials are riddled with pore structures, and show high adsorption capacity.

The layered double hydroxides (LDHs) had been widely used as environmental remediation materials for anions because of their outstanding ion exchange capacities^20,21^ and catalytic potentials^22,23^. LDHs could be expressed as: $\left[ {M_{1 - x}^{II} M_{x}^{III} \left( {OH^{ - } } \right)_{2} } \right]^{x + } \left( {A^{n - } } \right)_{{{x \mathord{\left/ {\vphantom {x n}} \right. \kern-\nulldelimiterspace} n}}}\cdot mH_{2} O$, where A^n-^ represents an anion in the interlayer; M^*II*^ and M^*III*^ represent the bivalent and trivalent metals, respectively; and x represents the molar ratio of ${{M^{III} } \mathord{\left/ {\vphantom {{M^{III} } {\left( {M^{II} + M^{III} } \right)}}} \right. \kern-\nulldelimiterspace} {\left( {M^{II} + M^{III} } \right)}}$ whose value is usually between 0.25 and 0.33^24^. Layered double hydroxides have good adsorption ability on both anions and cations due to their unique structural characteristics.

Although it has been reported that FeMn-LDH has good adsorption capacity as well as catalytic potential^24^, however, it’s difficult to obtain well layered FeMn-LDH materials by the common co-precipitation, and there are few studies on its preparation. A hydrotalcite-like absorbent named FeMgMn-LDH was synthesized via a co-precipitation method, and was proved to have good performance in heavy metals elimination in our previous study^25,26^. At room temperature, the FeMnMg-LDH can rapidly remove Cd^2+^ and Pb^2+^ ions, and the adsorption equilibrium can be reached within 120 min. The maximum adsorption capacities of Cd^2+^ and Pb^2+^ ions on FeMnMg-LDH were about 59.99 mg/g and 421.42 mg/g, respectively, which is significantly higher than that of other similar kinds of absorbents. However, its adsorption ability on anions has not been studied. At present, we further examined its adsorption characteristics for nitrate ions. BET, PXRD, FTIR, SEM–EDX and TEM were all used for characterization analysis. The influence of ion concentrations, temperature, pH, and coexisting anions, on nitrate ions removal by FeMgMn-LDH, were all considered in this study.

## Materials and methods

### Synthesis of FeMgMn-LDH

FeMgMn-LDH sample was obtained by a co-precipitation process^25^. Briefly, a mixed metal solution was obtained by dissolving FeCl_3_· 6H_2_O, MnCl_2_· 4H_2_O and MgCl_2_•6H_2_O into oxygen-free deionized water with an Fe^3+^, Mg^2+^, Mn^2+^ molar ratio of 1:2:1 and 2 mol/L metal ions in total. NaOH (analytical reagent) was put into the oxygen-free deionized water to obtain an alkaline solution. The mixed metal solution was dropped into a mixed flow reactor at a speed of 0.5 ml/min, and an alkaline solution was dropped in to maintain the pH around 10.0 at the same time. Co-precipitation was performed at room temperature under a nitrogen atmosphere and intense stirring. The reaction mixture was then fully agitated for 120 min at 25 ∘C and aged in the mother liquid for 24 h. The suspension was centrifuged and filtered, and the resulting solid was repeatedly washed and then freeze-dried.

### Characterization of FeMgMn-LDH

The cations in real water and in the adsorption samples were examined by an Agilent 7500a ICP-MS instrument. Phosphate ions were detected with a UV–vis spectrophotometer (Agilent Cary 60) through the molybdenum blue method. The specific surface area was analyzed by a Brunauer-Eett-Teller (BET) method with a Micromeritics ASAP-2010C automatic analyzer (Micromeritics Col Inc., Australia). PXRD patterns of the samples were recorded in a MIXima XRD-7000 diffractometer between 10° and 70° (2θ) using Cu*K*_α_ radiation. FTIR spectra of samples were determined by a Nicolet Magna 550 FT-IR instrument in the KBr phase. SEM images and EDX of samples were examined with an SU8020 microscope and EDAX instruments, respectively (Hitachi, Japan). TEM images were determined using a JEM 1200EX transmission electron microscope (JEOL, Japan).

### Removal studies

KNO_3_ (guarantee reagent) was dissolved into deionized water to prepare the nitrate stock solutions. During the adsorption study, a 0.45-μm microfiltration membrane was used for solid–liquid separation through a filter. Nitrate ions in the supernatant were detected with a Shimadzu UVmini-1240 spectrophotometer (Kyoto, Japan).

For the adsorption kinetics study, adsorbent was added into conical flasks with stoppers, separately. At specific intervals, the flasks were brought out and filtered promptly, to collect the filtrate. The filtrate was then detected using the methods mentioned above.

For equilibrium adsorption, the flasks containing reaction solutions (adsorbent dose = 1 g/L) were sealed and placed into a thermostatic shaker with a temperature of 25℃, shaken for 240 min at the speed of 120 rpm. In addition, a real sample of tail water from a domestic sewage treatment plant was used to test the nitrate ions removal ability of the adsorbent. The chemical composition of the real water was listed in Table S1 (Support information), and the other experimental factors were set as: adsorbent dose = 5 g/L; pH = 8.2; shaken speed = 120 rpm; and reaction time = 4 h at 25℃.

For the adsorption–desorption experiment, adsorption conditions are similar to the kinetics study (reaction time = 4 h). After adsorption, the adsorbent on membrane was washed to a conical flask for desorption test. A neutral solution containing 0.5 M NaCl was used for the nitrates desorption, and conditions were set as: adsorbent dose = 1 g/L; pH 7; shaken speed = 120 rpm; and reaction time = 6 h. After desorption, the adsorbent was washed to a conical flask for another round of adsorption–desorption experiment. The adsorption–desorption experiment was carried out in three rounds.

The pH of solutions was adjusted by 0.005–0.1 M HCl and NaOH solutions. The effects of coexisting anions including SO_4_^2−^, PO_4_^3−^ and CO_3_^2−^ were all examined at pH = 7, the other experimental factors were set as the conditions mentioned above.

## Results and discussions

### LDH characterizations

The Fe/Mg/Mn ratios in FeMgMn-LDH and Nitrate-FeMgMn-LDH were shown in Table 1. A clean and stable preparation process was assured because the molar ratio of Fe/Mg/Mn in FeMgMn-LDH was approximately equal to that in the mixed metal solution, which was used for co-precipitation. The molar ratio of Fe/Mg/Mn in Nitrate-FeMgMn-LDH was approximately equal to that in FeMgMn-LDH, indicating that there’s little influence in the layered structure by the nitrate adsorption. The lower percentage of metal ions in Nitrate-FeMgMn-LDH than that in FeMgMn-LDH was due to the weight increases caused by the ion exchange between Cl^−^ and NO_3_^−^.

**Table 1:** Fe:Mg:Mn ratio and lattice parameters of samples.

Sample	Elemental composition (wt%)			Fe:Mg:Mn ratio		Lattice parameters		Interlayer space (nm)
	Fe	Mg	Mn	In solution	In hydroxide	a (nm)	c (nm)	
FeMgMn-LDH	15.6	13.2	16.5	1:2:1	1:1.95:1.08	0.310	2.35	0.302
Nitrate-FeMgMn-LDH	15.2	12.7	16.2	–	1:1.94:1.08	0.310	2.33	0.297

The curves of N_2_ adsorption–desorption and pore size distribution are depicted in Fig. S1 (Support information). From the pore size distribution curve, the main pore diameter of FeMgMn-LDH is distributed in the range of 7 nm to 23 nm, and average pore diameter is about 38.21 nm. The specific surface area of FeMgMn-LDH is 47.17 m^2^/g, which is lower than that of other similar materials such as Mg_4_Al-LDH (64.4 m^2^/g)^27^, Mg_3_Fe-LDH (70 m^2^/g)^28^ and Mg_3_Al_0.1_Fe_0.9_-LDO (141.636 m^2^/g)^8^.

**Figure 1:**
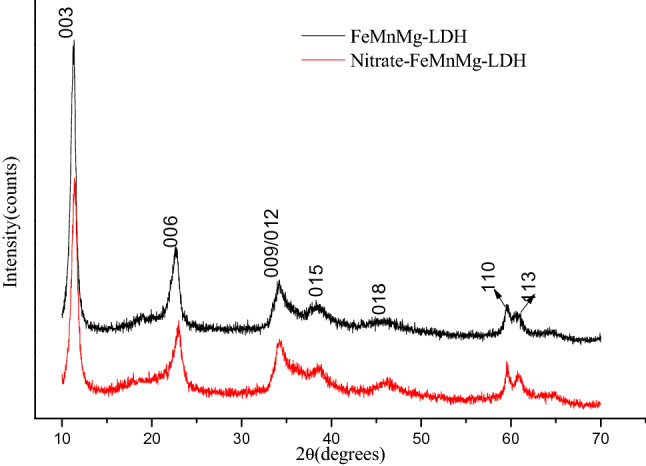
PXRD patterns of FeMgMn-LDH and nitrate-FeMgMn-LDH.

The PXRD patterns of FeMgMn-LDH and Nitrate-FeMgMn-LDH were depicted in Fig. 1. For FeMgMn-LDH, the typical structure of LDHs can be identified by the characteristic peaks: an intense peak (003) at 11.30° (7.82 Å) followed by two smaller peaks (006 and 009/012) at 22.80° and 34.19°, respectively, and two small peaks (110, 2θ = 59.63°and 113, 2θ = 60.82°) falling in the range of 59°-61°. For the Nitrate-FeMgMn-LDH, the typical characteristic peaks of LDHs still existed after adsorption, indicating that the typical lamellar structure of LDHs remained after nitrate adsorption. The location of the characteristic peaks had shifted to varying degrees, resulting from nitrate adsorption.

All the lattice parameters were listed in Table 1. The parameter “a” could be obtained from equation a = 2d_(110)_, which represents the ratio between bivalent and trivalent metal ions of the LDHs^25^. The basal spacing d_(003)_ is the sum of the brucite-like sheet (4.8 Å) and the interlayer space^29^. The interlayer space of the as-prepared FeMgMn-LDH is 3.09 Å, and that for their adsorption product (Nitrate-FeMgMn-LDH) is 2.97 Å. Additionally, the parameter “c” could be calculated from the equation c = 3d_(003),_ which is relevant to the interlayer space^30^, its value decreased about 0.2 Å after nitrate adsorption was caused by nitrate intercalation^28^.

FTIR spectra of FeMgMn-LDH and Nitrate-FeMgMn-LDH were depicted in Fig. S2 (Support information). As for the FeMgMn-LDH, the spectrum shows a broad at around 3400 cm^-1^ is assigned to -OH stretching vibration of the interlayer water or structural –OH groups^31^. The band at around 1620 cm^−1^ is in accord with –OH bending vibration of water molecules. The intense band at 1387 cm^−1^ in the spectrum of Nitrate-FeMgMn-LDH is assigned to N=O stretching vibration^8,18,32^, indicating that nitrate ions have intercalated into the interlayer of FeMgMn-LDH. The bands at around 590 and 420 cm^−1^ are in accord with lattice vibrations: for example M–O, M–OH and M–O–M groups (where M = Mg, Fe, and Mn) in the sheets of FeMgMn-LDH^33^.

**Figure 2:**
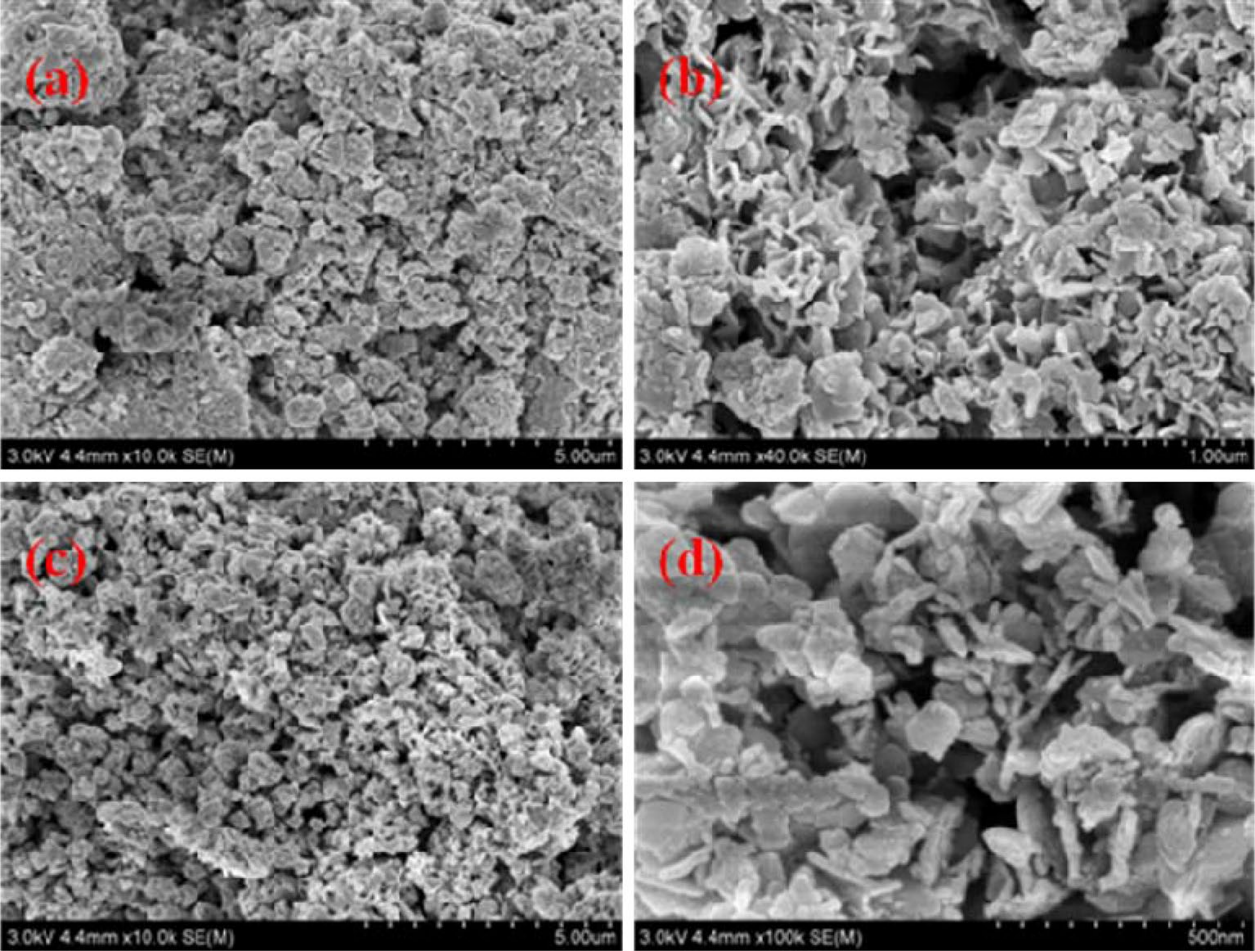
SEM photographs of samples: FeMgMn-LDH **(a, b)**; nitrate-FeMgMn-LDH **(c, d)**.

**Figure 3:**
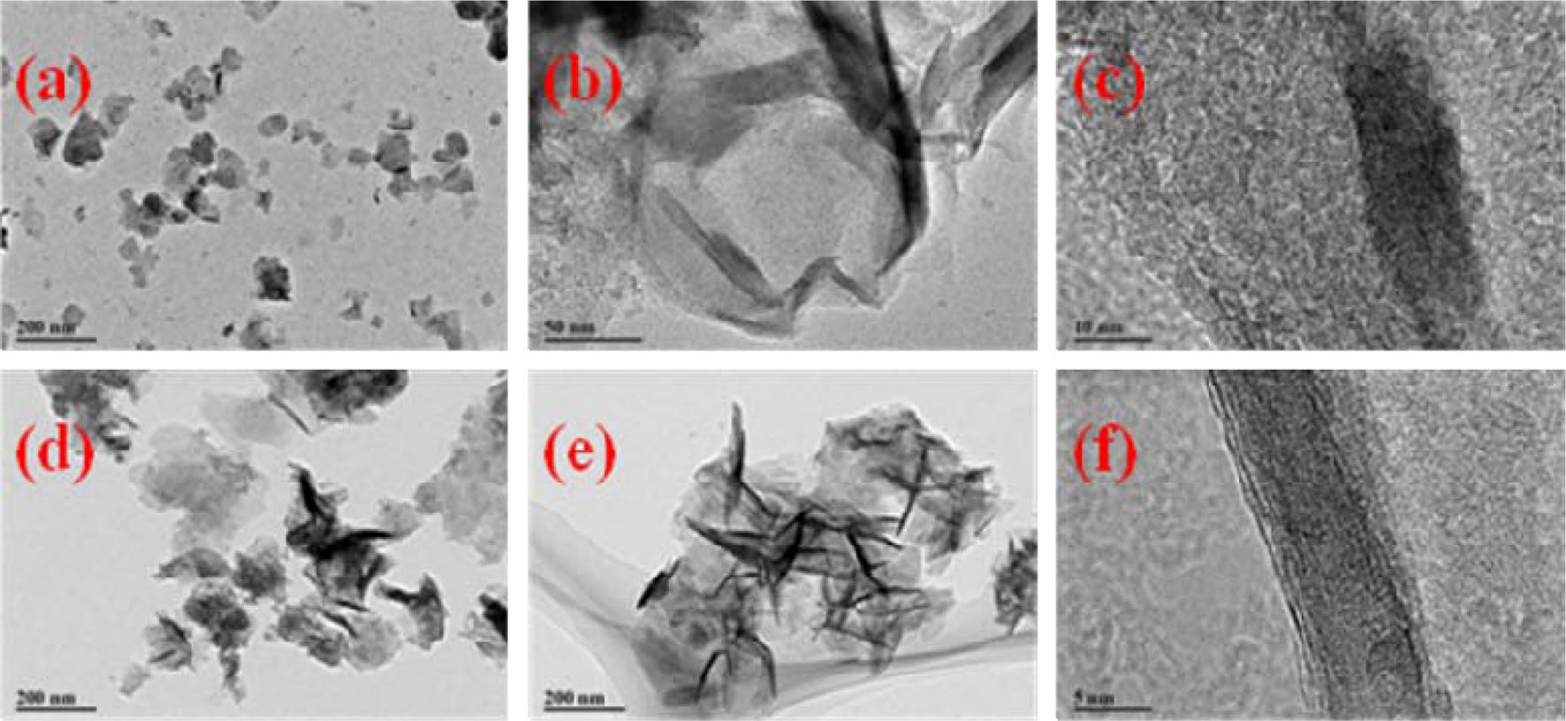
TEM photographs of samples: FeMgMn-LDH **(a–c)**; nitrate-FeMgMn-LDH **(d–f)**.

The SEM images of FeMgMn-LDH samples were depicted in Fig. 2. The particle shape of a hexagonal platelet can be clearly observed. The TEM images were depicted in Fig. 3. The relatively uniform layer, composed of thin nanoscale curved platelets with an average size of 100 nm, constitutes the FeMgMn-LDH, proving that the samples possess a hydrotalcite-like structure. Broken platelets and a fuzzy lattice fringe indicate poor crystallinity.

### Adsorption kinetics

The kinetic graphs of nitrate adsorption were depicted in Figs. S3 and S4 (Support information). As shown, the adsorption equilibrium can be reached within 120 min. Two generally applied kinetic models including the pseudo-first-order and pseudo-second-order kinetic models were used for fitting the experimental data.

1 $$\ln \left( {Q_{e} - Q_{t} } \right) = \ln Q_{e} - k_{p1} t$$

2 $$\frac{t}{{Q_{t} }} = \frac{1}{{k_{p2} Q_{e}^{2} }} + \frac{t}{{Q_{e} }}$$

In addition, the intra-particle diffusion model was applied to describe the adsorption kinetic as well:

3 $$Q_{t} = k_{3} t^{{{1 \mathord{\left/ {\vphantom {1 2}} \right. \kern-\nulldelimiterspace} 2}}}$$

where *k*_*p1*_, *k*_*p2*_ and *k*_*3*_ are the rate constants of each model, respectively, and *Q*_*t*_ and *Q*_*e*_ represent the quantity of adsorbate adsorbed by the adsorbent at moment t and equilibrium, respectively.

Usually, mass transfer is the first step in diffusion model: an adsorbate molecule was translated to the adsorbent surface from the aqueous solution. Intra-particle diffusion (or diffusion) is the second step: the adsorbate molecule is diffused onto the adsorption site within the particles, through either pore diffusion or a solid-surface diffusion mechanism. If there is a nonlinear relationship between *Q*_*t*_ and *t*^1/2^, intra-particle diffusion should be the speed-restricting step^34^.

The relevant parameters were shown in Table 2. Clearly, the fitting degree of pseudo-second-order function is higher than that of the other two models according to the R^2^ values, indicating that ion exchange might occur in the internal space^27^ rather than in the external space. According to value of k_3_ and k_3_’, its not difficult to find that the moving speed of the adsorbate molecule in the second step is obviously slower than that in the first step (Fig. S4 (Support information)), indicating that diffusion is the speed-restricting step of nitrate adsorption, and during which ion exchange must be occurred.

**Table 2:** Kinetics constants of nitrate ions adsorption.

Sample	*Q*_e,exp_ (mg g^−1^)	Pseudo-first-order function			Pseudo-second-order function			Intra-particle diffusion model			
		*Q*_e,cal_ (mg g^−1^)	*kp*_1_ (min^−1^)	*R*^2^	*Q*_e,cal_ (mg g^−1^)	*kp*_2_ (g mg^−1^ min^−1^)	*R*^2^	k_3_ (mg g^−1^ min^−1/2^)	R^2^	k_3_´ (mg g^−1^ min^−1/2^)	R^2^
FeMgMn-LDH	4.50	4.38	0.266	0.937	4.65	0.0882	0.985	0.982	0.958	0.040	0.966

### Adsorption isotherms

The isothermal adsorption curves were depicted in Fig. S5 (Support information). Besides the Langmuir and Freundlich isotherm models, the Dubinin-Kaganer-Radushkevich model was also applied to fit the adsorption data, which is presented as follow:

4 $$\ln Q_{e} = \ln Q_{m} - \beta \varepsilon^{2}$$

where *Q*_e_ and *Q*_max_ represent equilibrium adsorption capacity and maximum adsorption capacity in theory, respectively; the activity coefficient β (mol^2^ J^−2^) stands for the average adsorption energy; ε stands for the Polanyi potential can be expressed as follow:

5 $$\varepsilon = RT\ln \left( {1 + \frac{1}{{C_{e} }}} \right)$$

where T and R represent the Kelvin’s temperature (K) and the ideal gas constant, respectively. The adsorption space around the adsorbent surface consists of equipotential surfaces (with equal adsorption potential)^25^. E stands for the apparent adsorption energy can be expressed as follow:

6 $$E = \frac{1}{{\sqrt { - 2\beta } }}$$

Generally, the adsorption mechanism can be educed from the distribution of E value. A value lower than 8 (kJ/mol) may imply a physical adsorption; one falling into the range of 8–16 (kJ/mol) suggests an ion exchange; and a value higher than 16 (kJ/mol) indicates that the mechanism might be chemical adsorption^35^.

According to Table 3, the high correlation coefficients R^2^ verified the consistency between theoretical models and experimental data. The good agreement between experimental data and Freundlich model represents that the nitrate adsorption by FeMgMn-LDH is not limited by the external surfaces, but by the internal surface. The adsorption capacity of calcined LDHs to anions is higher than that of their primary LDHs, which is related to the "memory effect". During the restoration of the layered structure, anions can be inserted into the interlayer of LDHs and removed from the solution. The maximum amount of nitrate adsorption on FeMgMn-LDH at 25℃ was 10.56 N-mg/g. Although its lower than that of the calcined LDHs and modified LDHs, but its higher than that of other primary LDHs (Table 4), which may due to the nitrate-selective anion exchange capacity of LDHs^30^, indicating that FeMgMn-LDH has the potential to be used as nitrate adsorbent.

**Table 3:** Constants for adsorption isotherm models.

Temperature (K)	*Q*_*exp*_ (mg g^-1^)	Langmuir model			Freundlich model			DKR model			
		*k*_*l*_ (L mg^−1^)	*Q*_*m,cal*_ (mg g^−1^)	*R*^2^	*k*_*f*_ ((L mg^−1^)^1/n^ mg g^−1^)	n	*R*^2^	*Q*_*m,cal*_ (mg g^−1^)	*Β* (mol^2^ J^−2^)	*E* (kJ mol^−1^)	*R*^2^
288.15	10.58	0.062	12.55	0.996	1.62	2.30	0.982	26.31	− 5.84 × 10^–9^	9.249	0.997
298.15	9.02	0.057	10.56	0.996	1.29	2.26	0.980	22.84	− 5.70 × 10^–9^	9.363	0.997
308.15	7.22	0.052	8.57	0.996	0.97	2.20	0.979	18.81	− 5.54 × 10^–9^	9.500	0.997

**Table 4:** Nitrate adsorption parameters of different LDHs.

Material	Adsorption capacity (mg/g)	T (°C)	pH	Ref
Mg_4_Fe-Cl-LDH	8.71	25	7	27
Mg_4_Fe-Cl-LDH	7.69			
Mg_3_Al-Cl-LDH	8.97			
500-Mg_2_Al-Cl-LDO	11.93	25	7.1	2
500-Mg_3_Al-Cl-LDO	26.95			
500-Mg_4_Al-Cl-LDO	34.36			
Biochar/MgFe-LDH	24.8	22 ± 0.5	–	3
NiFe-Cl-LDH	2.35	RT	–	39
450-Co_1.5_Mg_1.5_Al-LDO	12.2	30	7	8
450-Co_2.5_Mg_0.5_Al-LDO	5.63			
450-Co_1.5_Mg_1.5_Fe-LDO	4.69			
450-Cu_1.5_Zn_1.5_Al-LDO	1.43			
450-Mg_3_Al_0.1_Fe_0.9_-LDO	123.3			
MgAl-CO_3_-LDH	0.72	25 ± 2	6	18
ZnCr-Cl-LDH	2.24			
MgMn-Cl-LDH	0.76			
MgCr-Cl-LDH	1.96			
CaCr-Cl-LDH	1.04			
FeMgMn-LDH	10.56	25	7	This study

The value of the Freundlich parameter n is 2.26, which falls between 2 and 10, indicating a favorable adsorption; k_f_ is 1.29 and the Langmuir constant k_l_ is 0.057. While the values of the DKR parameters Q_m_, β and E are 22.84 N-mg/g, − 5.70 × 10^−9^ mol^2^/J^2^ and 9.363 kJ/mol, respectively. It is clear that the values of E are in the range of 9–10 kJ/mol at the experimental temperatures, indicating that an ion exchange mechanism may predominate during the adsorption process.

According to the parameters shown in Table 3, the Q_exp_ for NO_3_^-^ removal on FeMgMn-LDH decreased when the experimental temperature increased from 288.15 K to 308.15 K. Thermodynamic parameters, including Gibbs free energy (ΔG^0^), entropy (ΔS^0^) and enthalpy (ΔH^0^), were used for describing this process. These parameters can be obtained according to the equations:

7 $$\Delta G^{0} = - RT\ln k_{d}$$

8 $$\ln k_{d} = \frac{{\Delta S^{0} }}{R} - \frac{{\Delta H^{0} }}{RT}$$

where the distribution ratio can be calculated from k_d_ = 10^6^ k_l_, when the adsorption data are in accordance with the Langmuir isotherm model^36,37^. As shown in Table 5, the positive value of ΔS^0^ (0.068 kJ mol^−1^ K^−1^) indicates that the disorder at the interface of solid-solution continued increase during the adsorption process. Meanwhile, the negative values of ΔG^0^ (from − 27.796 to − 26.426 kJ mol^−1^) and ΔH^0^ (− 6.678 kJ mol^−1^) suggest that the nitrate ions adsorption process on FeMgMn-LDH was spontaneous and exothermic.

**Table 5:** Thermodynamic constants of nitrate adsorption.

T(K)	lnk_d_	ΔG^0^ (kJ mol^−1^)	ΔH^0^ (kJ mol^−1^)	ΔS^0^ (kJ mol^−1^ K^−1^)	R^2^
288.15	11.030	− 26.426	− 6.678	0.068	0.995
298.15	10.948	− 27.139			
308.15	10.849	− 27.796			

### Nitrate adsorption in real water

**Figure 4:**
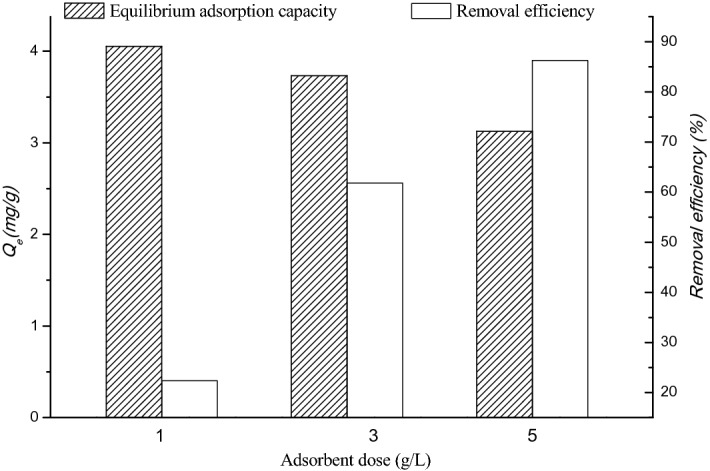
The adsorption efficiency of nitrate by FeMnMg-LDH in real water (initial pH 8.2).

The removal efficiency of nitrate ions was increased with the increasing dose of the adsorbent in the real water. The removal rate of nitrate ions was presented as 22.36%, 61.82% and 86.26% with the adsorbent dose of 1, 3 and 5 g/L, respectively (Fig. 4). Meanwhile, the equilibrium adsorption capacity was decreased along with the increasing dose of the adsorbent, and that was found to be 4.05, 3.73 and 3.13 N-mg/g, respectively. The heavy metal ions present in real water were completely eliminated, while the removal efficiency of phosphate was increased for 99.0%, 99.4% and 99.8%, depending on the increasing dose of the adsorbent of 1, 3 and 5 g/L, respectively.

### Adsorption–desorption experiment

The adsorption removal rates of nitrate ions were basically maintained at a certain level (Fig. S6 (Support information)), indicating that the main structure of FeMgMn-LDH remained intact during the adsorption–desorption rounds. The adsorbed nitrate ions could be desorbed completely in a high concentration of chloride ions atmosphere, indicating that the adsorption of nitrate ions is reversible. Thus, FeMnMg-LDH can be used as an alternative adsorbent for nitrate ions removal.

### Mechanism of adsorption by FeMgMn-LDH

Generally, LDHs remove anions from aqueous solution mainly through four mechanisms: (1) ion exchange with the interlayer anions; (2) electrostatic attraction by the positively charged LDH layers; (3) chelating with the metal ions in LDH layers and (4) the reconstruction of primary layered structures by the “memory effect”.

#### Effect of pH

**Figure 5:**
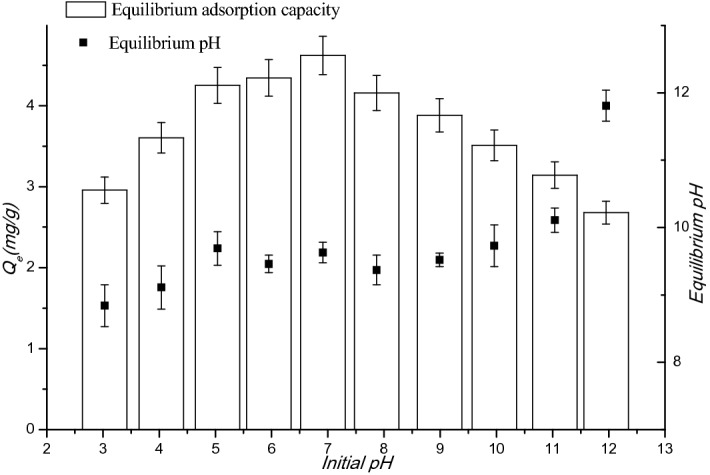
Effects of pH on the removal of nitrate (initial concentration = 20 mg/L).

It clear from Fig. 5 that the adsorption removal efficiency of nitrate increased along with the pH increase when initial pH is lower than 7, and decreased as pH increased, when the initial pH is higher. Meanwhile the maximum removal efficiency occurred in the range of pH 5–8, indicating that the electrostatic attraction mechanism may exist in the adsorption process:

9 $$NO_{3}^{ - } + \equiv MOH_{2}^{ + } \to \equiv MOH_{2} \cdots NO_{3}$$

After the adsorption of nitrate, the final pH values tended to fall between 9 and 10 (except that initial pH 12), resulting from the high buffer capacity^36^ of FeMgMn-LDH.

#### Effects of competitive ions

**Figure 6:**
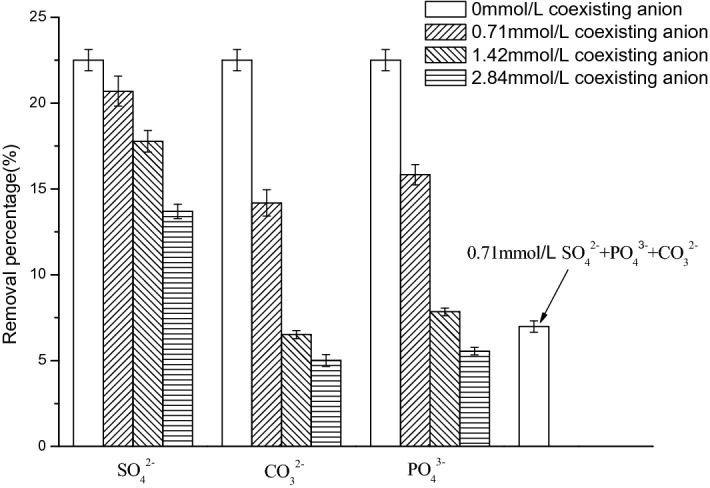
Effects of competitive anions on the adsorption of nitrate (pH 7; initial concentration = 20 mg/L).

Anions such as SO_4_^2−^, PO_4_^3−^ and CO_3_^2−^ usually coexist with nitrate ions in wastewater, and compete with the adsorption sites of adsorbent. Therefore, the effects of these competitive anions were investigated in this study. According to Fig. 6, when there were no other anions, the adsorption removal rate of NO_3_^−^ on FeMgMn-LDH was about 22.51%. The high concentration of coexisting anions may decrease the NO_3_^−^ removal efficiency sharply. When 2.84 mmol/L SO_4_^2−^ was added to the system, the removal rate of NO_3_^-^ decreased from 22.51 to 13.7%, while for CO_3_^2−^ and PO_4_^3−^, the NO_3_^−^ removal rates were 5.01% and 5.54%, respectively. The slight effect of SO_4_^2−^ on the NO_3_^−^ removal was attributed to the selective anion exchange ability of FeMgMn-LDH based on the anion-sieve effect, and similar results have been reported by others^30,38^. The strong effect of PO_4_^3−^ is more likely due to the electrostatic repulsion and strong interaction between the skeletal metals and PO_4_^3−^. Except for the electrostatic repulsion and chemical interaction, CO_3_^2−^ also competes with NO_3_^−^ for adsorption site through anion-sieve effect due to their similar ion diameter. Therefore, the competition order of coexisting anions on nitrate adsorption with FeMgMn-LDH is CO_3_^2−^ > PO_4_^3−^ > SO_4_^2−^.

**Figure 7:**
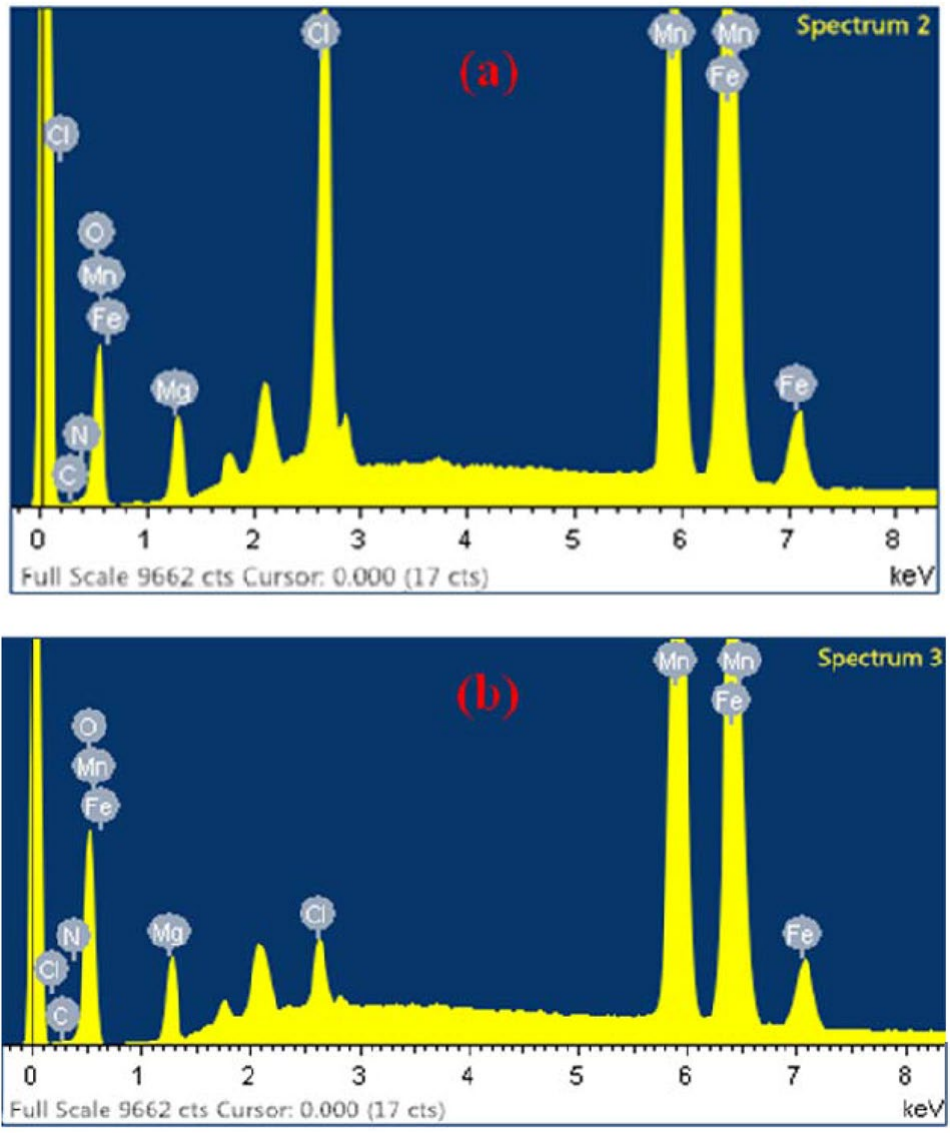
EDX analysis of sample before **(a)** and after **(b)** nitrate adsorption.

From the EDX analysis of FeMgMn-LDH and Nitrate-FeMgMn-LDH (Fig. 7), it can be seen that the intensity of the Cl peak, which represents the content of Cl^-^ ions in the FeMgMn-LDH sample, dropped rapidly after the nitrite was adsorbed, meanwhile, the amount of Cl was reduced from 6.18% (in FeMgMn-LDH) to 1.81% (in Nitrate-FeMgMn-LDH), indicating that ion exchange must have occurred between NO_3_^−^ and Cl^−^. In addition, the apparent adsorption energy (E) calculated from the DKR model provides further evidence that the ion exchange mechanism may predominate the adsorption process, and it can be presented as:

10 $$NO_{3 {{\left( {aq} \right)}}}^{ - } + \equiv Cl^{- }_{\left( L \right)} \to \equiv NO_{3{\left( L \right)}}^{ - } + Cl^{ - }_{{\left( {aq} \right)}}$$

Thus, the removal of nitrate on FeMgMn-LDH in this study is mainly by ion exchange and electrostatic attraction.

## Conclusions

It is shown that in this study the prepared FeMgMn-LDH is a promising adsorbent for nitrate removal, which has high buffer capacity (final pH remained between 9 and 10) and high reversibility. Adsorption equilibrium could be well fitted by the three isotherm models, and the maximum amount of nitrate adsorption at 25 ℃ is 10.56 N-mg g^−1^. In a real water, the removal rate of nitrate ions can reach 86.26% with the adsorbent dose of 5 g/L. There is a nonlinear relationship between *t*^1/2^ and *Q*_*t*_, which indicates that intra-particle diffusion is the speed-restricting step. Moreover, the values of apparent sorption energy within the range of 9–10 kJ/mol at the experimental temperatures and the change of Cl content shown in EDX analysis both indicate that an ion exchange mechanism may predominate adsorption process. The results of adsorption–desorption experiments show that FeMgMn-LDH has good adsorption capacity for nitrate ions. The effects of three coexisting anions on nitrate ions removal differ, following the order CO_3_^2−^ > PO_4_^3−^ > SO_4_^2−^. FeMgMn-LDH can remove nitrate ions selectively though an anion-sieve effect which is mainly controlled by physical. The positive value of ΔS^0^ (0.068 kJ mol^−1^ K^−1^) indicates a continued increase of disorder at the interface of the solid-solution. Meanwhile, the negative values of ΔG^0^ (from − 27.796 to − 26.426 kJ mol^−1^) and ΔH^0^ (− 6.678 kJ mol^−1^) suggest that the nitrate ions adsorption process on FeMgMn-LDH is spontaneous and exothermic. The main adsorption mechanisms involve electrostatic attraction and ion exchange.

## Supplementary information

Supplementary Information.
