# Supplementary material for: Selective nitrate removal from aqueous solutions by a hydrotalcite-like absorbent FeMgMn-LDH
Source: Sci Rep. 2020 Sep 30;10:16126. doi: 10.1038/s41598-020-72845-3 (PMC7528107; doi:10.1038/s41598-020-72845-3)
Supplement: Supplementary file 1 — Supplementary Information. [file 41598_2020_72845_MOESM1_ESM.doc]

Supplementary Material for

**Selective nitrate removal from aqueous solutions by a hydrotalcite-like absorbent FeMgMn-LDH**

Hongguang Zhou^1^, Youlin Tan^1^, Wei Gao^1^, Yue Zhang^2^ and Yanmei Yang^1^

^1^National Engineering Research Center for Inland Waterway Regulation, Chongqing Jiaotong University, Chongqing 400074, China

^2^ North China Municipal Engineering Design and Research Institute Co. Ltd., Tianjin 300074, China

Corresponding author at: National Engineering Research Center for Inland Waterway Regulation, Chongqing Jiaotong University, Chongqing 400074, China

E-mail address: yymeicq@163.com (Y. Yang)

**Table S1** The chemical composition of the real water.

| Composition | Concentrations |
| --- | --- |
| pH | 8.2 |
| COD | 8 (mg/L) |
| BOD_5_ | 1.2 (mg/L) |
| Nitrate | 18.12 (mg/L) |
| Phosphate | 0.66 (mg/L) |
| Cadmium | 0.0008 (mg/L) |
| Chromium | 0.00314 (mg/L) |
| Arsenic | 0.0004 (mg/L) |
| Lead | 0.0011 (mg/L) |
| Mercury | 0.0003 (mg/L) |

**Fig. S1.** N_2_ adsorption-desorption isotherm and pore size distribution of FeMgMn-LDH

**Fig. S2**. FTIR spectra of samples: (1) FeMgMn-LDH; (2) Nitrate-FeMgMn-LDH

**Fig. S3.** Kinetic graphs of nitrate ions adsorption (initial concentration =20 mg/L and pH =7)

**Fig. S4.** Intra-particle diffusion kinetic of nitrate ions adsorption

**Fig. S5.** Experimental data (a) and their model fittings by Langmuir (b), Freundlich (c) and DKR (d) models (initial pH =7)

**Fig. S6.** The adsorption-desorption efficiency of nitrate by FeMnMg-LDH
